# Gangrene of the Lungs Successfully Treated by Inhalations of Terebinthinate Vapors

**Published:** 1854-11

**Authors:** 


From the Lancet.

### *Gangrene of the Lung successfully treated by Inhalations of Terebinthinate Vapors.*

Dr. Skoda has published, in the Zeitchschrift, &c. of Vienna, several cases of gangrene of the lung, in which the symptoms gave way by the use of terrebinthinate vapors and the administration of quinine. In the first case, the cure was effected in six weeks upon a servant, with whom the gangrene had attacked the upper lobe on the right side. An inn keeper, of middle age, was equally benefited by the same means, but the cure took a longer time and a stay in the country. He also took one grain of quinine every second hour. The treatment was not properly carried out in the third case; and the fourth, that of a journeyman butcher, of a robust constitution, is still pending. The latter had, however, so far recovered, after using the inhalations, and also taking Fowler's solution, that he could go into the country, though there was still some uneasiness in the left scapular region. The inhalations are made by pouring oil of turpentine on boiling water, the inspirations being repeated every second hour, and carried on for fifteen minutes.

